# Limited plane analysis reduces accuracy of 3-dimensional reconstruction of the right ventricle

**DOI:** 10.1186/1532-429X-11-S1-P81

**Published:** 2009-01-28

**Authors:** Florence Sheehan, Ruslan Kazakov, Mary-Pierre Waiss

**Affiliations:** 1 University of Washington, Seattle, WA USA; 2 VentriPoint, Inc, Seattle, WA USA

**Keywords:** Congenital Heart Disease, Right Ventricle, Right Ventricle Ejection Fraction, Right Ventricle Volume, View Subset

## Background

Ventricular remodeling is a compensatory process that is little understood in the right ventricle (RV). We have previously reported an association between abnormalities in RV shape and function in patients with dilated RVs. In these studies we used the piecewise smooth subdivision surface (PSSS) reconstruction method because it provides accurate representation of the 3D shape of the RV. However the complexity of RV shape mandates manual tracing of RV borders from many images.

## Methods

We tested the error in RV volume, ejection fraction (EF), and shape accrued by reducing the number of borders traced from MRI studies of 18 patients with congenital heart disease and 2 normal subjects. The true volume and shape were defined by tracing RV borders from 17 to 33 (mean 23 ± 4) images recorded in short axis (SAX), long axis (LAX), oblique, and inflow-outflow track (IOT) views. LAX views could be radially oriented about the left ventricular LAX or parallel to the 4-chamber view, but always visualized the entire free wall from its inferior to superior aspect. Reduced data sets were created containing 10, 12, 14, or 16 image planes distributed between SAX, LAX, and IOT views. Because pilot studies suggested that 12 might suffice, 3 subsets of 12 views were created that emphasized SAX views (12s), LAX views (12l), or the combination (12c). The RV was reconstructed at end diastole and end systole from full border sets and from each subset of borders using the PSSS method. Volume was computed directly from the 3D surface and used to calculate EF. Shape was measured at 20 cross sections from apex to tricuspid valve as eccentricity (= 4 piArea/Perimeter^2^).

## Results

RV volume at end systole was overestimated by 1.5 ± 3.0% (p < 0.05) using 10 and 12c subsets. RV EF was underestimated by 2–3% (p < 0.05) using 10, 12l, and 12c subsets. RV volume and EF did not differ significantly from true when measured using 12s, 14, or 16 view subsets. RV shape at end diastole but not at end systole was slightly but significantly more rounded in the two most apical slices when measured using 14 views. There was also small but significant underestimation at end diastole, although not at end systole, of other RV shape metrics: the ratio of septal area to total surface area, RV LAX length, and extent of bulging at the base. When reconstructed using 16 views, the only shape metric to show a significant difference from true was eccentricity at one slice.

## Conclusion

Accurate measurement of RV volume and EF can be obtained from the PSSS method after tracing 14 views comprising 6 SAX slices mainly concentrated at the base, 6 LAX views distributed evenly from inferior to superior aspects of the free wall, and 2 IOT views. For the detailed 3D shape analysis that PSSS reconstruction enables, a minimum of 16 views is recommended.

